# Editorial for Special Issue “Recombinant Proteins for Molecular Biology Research: Technologies and Applications”

**DOI:** 10.3390/cimb48060604

**Published:** 2026-06-07

**Authors:** Tsutomu Arakawa

**Affiliations:** Alliance Protein Laboratories, San Diego, CA 92121, USA; tarakawa2@aol.com

Proteins are extensively used for research, food processing and pharmaceuticals. These proteins can be prepared from natural sources, including plant seeds, cells, tissues, body fluids or even whole organisms, which are often encountered by contaminations, limited quantities or heterogeneity due to post-translational modifications [1,2,3,4,5]. Genetic engineering and recombinant technologies changed entirely the process of protein production and vastly improved efficiency, and the quantity and quality of protein production [6,7,8]. Once genes of interest become available, they are transfected into host cells or cell extracts using genetic engineering, recombinant technologies [9,10,11]. Various host cells or cell-free systems have been used for heterologous recombinant gene expressions, encompassing bacterial cells (mainly *Escherichia coli*), yeast cells, insect cells, mammalian cells and even whole plants or animals [12,13,14,15]. Each host expression system uses different vectors for gene delivery and has advantages and disadvantages in terms of expression level, scalability, downstream process and contamination level. For example, mammalian expression system can confer natural glycosylation for human-derived glycoproteins and is primarily used for producing recombinant therapeutic proteins. Bacterial expression is inexpensive and can produce a large quantity but may require refolding that is still based on trial-and-error experiments. They may be a natural form or fused to a protein or a peptide tag to augment the therapeutic efficacy of the natural protein. Fusion technology is also used to facilitate purification of proteins for both research and pharmaceutical applications. This special issue covers various topics related to production of recombinant proteins. First, fundamental aspects of protein folding and biological interactions and the effects of small molecule additive to manipulate protein solutions are covered by Arakawa et al. [16]. The review describes how protein folding leads to presentation of amino acid side chains that offer biologically relevant molecular interactions. Many different utilities of fusion-tags were presented by Arakawa and Akuta [17]. Fusion-tags have been originally developed to facilitate purification. The review summarizes many potential applications of fusion-tags, including increasing soluble expression, altering pharmacological functions, and other biological utilities. Among various fusion-tags, SUMO-tag has been shown effective in enhancing soluble expression [18,19]. Granulocyte colony-stimulating factor (GM-SCF) stimulates production of hematopoietic cells [20]. Volosnikova et al. [21] used SUMO-tag to enhance prokaryotic expression of GM-CSF and assist refolding. Sasaki et al. [22] attempted to enhance antigenic response of IGG production against coronavirus 2 by a fusion with a nasal immuno-inducible sequence. *Mycoplasma hyopneumoniae* infection severely affects the mortality of pigs, causing significant economic losses to the pig industry. CD40L is a molecular adjuvant that enhances the cellular and humoral immune responses to vaccines. Shu et al. [23] fused the CD40L peptide to the C-terminus of the chimeric antigenic vaccine protein to potentiate the humoral and cellular immune responses to the infection in the mouse model. Gad et al. [24] successfully expressed a catalytic domain of an industrial enzyme, L-gulonolactone oxidase enzyme, in *Escherichia coli*. The L-gulonolactone oxidase enzyme catalyzes the last step of L-ascorbic acid (vitamin C) biosynthesis. Byambaragchaa et al. [25] used a CHO cell expression to express a tethered eel luteinizing hormone, resulting in a highly potent hormone analog. Recombinant enzyme used to process food products was successfully produced on cell membranes of *Escherichia coli.* Muñoz-Muñoz et al. [26] developed expression of *Escherichia coli* phytase, widely used as an exogenous enzyme in monogastric animal feed, mainly because of its ability to degrade phytic acid or its salt form (phytate), a natural source of phosphorus. They explored a homologous protein production approach for displaying the enzyme on the cell surface of *Escherichia coli* by engineering its outer membrane (OM) for extracellular expression. Liu et al. [27] used a cell-free protein synthesis system based on wheat germ extract. To overcome the energy limitation common in such systems, they engineered an *Escherichia coli* strain to function as a self-renewing ATP source. This strain co-expresses a three-enzyme cascade—adenosine kinase, adenylate kinase, and acetate kinase—that efficiently converts adenosine and acetyl phosphate into ATP. Using the lysate from this biocatalyst to energize an optimized wheat germ extract, a high-performance cell-free synthesis platform was established. This integrated system supported the robust production of multiple recombinant proteins.
